# “Real‐world” eligibility for anti‐amyloid treatment in a tertiary memory clinic setting

**DOI:** 10.1002/alz.70375

**Published:** 2025-06-12

**Authors:** Sinthujah Vigneswaran, Everard G. B. Vijverberg, Frederik Barkhof, Elsmarieke van de Giessen, Afina W. Lemstra, Yolande Pijnenburg, Charlotte E. Teunissen, Wiesje M. van der Flier, Argonde C. van Harten

**Affiliations:** ^1^ Neurochemistry Laboratory Department of Laboratory Medicine Amsterdam UMC, Vrije Universiteit Amsterdam, Amsterdam Neuroscience Amsterdam the Netherlands; ^2^ Alzheimer Center Department of Neurology Amsterdam UMC, Vrije Universiteit Amsterdam, Amsterdam Neuroscience Amsterdam the Netherlands; ^3^ Amsterdam Neuroscience Program Neurodegeneration Amsterdam the Netherlands; ^4^ Department of Radiology and Nuclear Medicine Amsterdam UMC, Vrije Universiteit Amsterdam Amsterdam the Netherlands

**Keywords:** Alzheimer's disease, anti‐amyloid treatment, eligibility, real‐world evidence

## Abstract

**INTRODUCTION:**

The societal impact of anti‐amyloid treatment (AAT) for Alzheimer's disease (AD) depends largely on patient eligibility. Estimates suggest that 1% to 18% of individuals with AD may qualify for AAT; however, data from everyday clinical practice remain limited. This study assessed AAT eligibility in patients at a tertiary memory clinic.

**METHODS:**

We included 1309 new patients (63 ± 8 years, 45% women, Mini‐Mental State Examination [MMSE] 25 ± 5) who presented to the Alzheimer Center Amsterdam (2020–2022) for standardized diagnostic workup. Eligibility for AAT was based on lecanemab's approved label guidelines.

**RESULTS:**

Of 1309 new patients, 514 (39% of new patients) had clinical mild cognitive impairment (MCI) or AD. Of these, 108 (8% new patients/21% clinical MCI/AD) met Clinical Dementia Rating/MMSE criteria, were amyloid positive, and had < 4 microbleeds/superficial siderosis. After further excluding apolipoprotein E ε4/ε4 homozygotes and anticoagulant users, 79 patients (6% new patients/15% clinical MCI/AD) remained eligible.

**DISCUSSION:**

Findings indicate limited eligibility for AAT in tertiary memory clinics.

**Highlights:**

- Initial eligibility for lecanemab was 8% of all patients and 21% of those with clinical mild cognitive impairment (MCI) or Alzheimer's disease (AD) based on approved guidelines in a tertiary memory clinic population.
- After strict exclusions (such as apolipoprotein E ε4/ε4 homozygosity and anticoagulant use), eligibility dropped to 6% of all patients and 15% of those with clinical MCI or AD.
- The study highlights the limited real‐world applicability of anti‐amyloid treatment under current guidelines.

## 1 INTRODUCTION

Anti‐amyloid therapy (AAT) for Alzheimer's disease (AD) has recently advanced in a major way, with several therapies gaining regulatory approval across the globe. These therapies are now approved for individuals with mild cognitive impairment (MCI) or mild dementia due to AD in countries including the United States, United Kingdom, Japan, China, and Europe.^1^, ^2^, ^3^, ^4^, ^5^, ^6^ As treatment for AD becomes available, understanding the number of potentially eligible patients grows increasingly important, especially given the substantial costs associated with these drugs.^7^, ^8^ Population‐based estimates suggest that 1% to 18% of memory‐clinic attendees or community‐dwelling population would meet AAT criteria based on abnormal cerebrospinal fluid (CSF) or amyloid positron emission tomography (PET) and the European Alzheimer's Disease Consortium projected that up to one third of amyloid‐positive MCI patients across 27 European countries could be eligible for lecanemab.^7^, ^9^, ^10^, ^11^, ^12^, ^13^, ^14^, ^15^, ^16^ However, these figures are derived from prevalence data and modeling rather than from direct, routine biomarker assessments, leaving a gap in real‐world evidence.^17^ To address this, we reviewed all consecutive patients seen in our tertiary memory clinic between 2020 and 2022 and systematically applied the current lecanemab appropriate‐use criteria to determine the actual proportion eligible for AAT.^18^, ^19^

## 2 METHODS

### 2.1 Participants

Between January 1, 2020, and December 31, 2022, we enrolled all consecutive patients (*n* =  1309) who sought medical care at the Alzheimer Center Amsterdam memory clinic for evaluation of cognitive symptoms and provided informed consent for research use of their medical data. Patients underwent a standardized diagnostic workup consisting of a visit to a neurologist, including physical and neurological examination during which medical history and current medications were systematically recorded; standardized neuropsychological testing; brain magnetic resonance imaging (MRI); laboratory investigations; and CSF biomarker testing as described elsewhere.^20^, ^21^ Some patients underwent amyloid PET imaging either as part of research protocols or for diagnostic purposes.^20^ All brain MRI examinations were performed on a 3 T scanner using standard gradient‑echo sequences with 5 mm slice thickness. The clinical diagnoses were made in a multidisciplinary consensus meeting based on applicable criteria for MCI, AD dementia, frontotemporal dementia, and dementia with Lewy bodies.^22^, ^23^, ^24^, ^25^ Participants experiencing cognitive complaints with normal clinical and cognitive test results who did not meet the criteria for MCI, dementia, or other neurologic or psychiatric conditions were labelled as subjective cognitive decline.^26^

### 2.2 Amyloid status

Amyloid status was based on the CSF phosphorylated tau (p‐tau)181/amyloid beta (Aβ)1‐42 ratio (*n* = 869) or amyloid PET imaging (*n* = 96). Patients who underwent an amyloid PET scan were scanned with [^18^F]florbetaben, [^18^F]florbetapir, [^18^F]flutemetamol, or [^11^C]Pittsburgh compound B radiotracers. The PET scans were visually rated as amyloid positive or negative by an experienced nuclear medicine physician. CSF was collected by lumbar puncture in the L3/L4 or L4/L5 intervertebral space under non‐fasting conditions.^27^ CSF Aβ1‐42, total tau, and p‐tau181 values were measured using Elecsys electrochemiluminescence immunoassays (Roche Diagnostics International Ltd.). The threshold for Elecsys P‐tau181/Abeta42 is > 0.02.^28^ If both CSF and PET were available, we used the PET outcome to determine amyloid status. There was no amyloid status available for 25 (5%) of clinical MCI/AD patients.

### 2.3 Eligibility for AAT

The criteria we applied to determine eligibility for AAT were in accordance with the US Food and Drug Administration–provided prescribing information for lecanemab and the appropriate use criteria for lecanemab.^18^, ^19^ Patients were considered eligible for AAT based on (1) a primary clinical diagnosis of MCI or AD dementia with, (2) Clinical Dementia Rating (CDR) of 0.5 or 1.0 and a Mini‐Mental State Examination (MMSE) ≥ 22 and ≤ 27, (3) amyloid positivity, and (4) < 4 microbleeds or superficial siderosis on brain MRI. In the final step, we excluded patients with an apolipoprotein E (*APOE*) ε4/ε4 genotype and those using anticoagulants, as these were considered relative contraindications and are now also part of the European Medicines Agency recommended criteria for the use of lecanemab.^29^

### 2.4 Statistical analysis

The descriptive statistics of the Amsterdam Dementia Cohort were performed in R language and environment for statistical computing version 4.0.3. Sociodemographic and medical characteristics associations were estimated using one‐way analysis of variance. A *p* value of < 0.05 was considered significant for baseline associations. For patients with clinical MCI or AD and unknown amyloid status (*n* = 25), we calculated a range, assuming all 25 could be either amyloid positive (upper limit) or amyloid negative (lower limit). After applying criteria for microbleeds/superficial siderosis, 13 patients remained eligible who could potentially be either amyloid positive or negative. After the exclusion of patients with an *APOE* ε4/ε4 genotype and those on anticoagulant therapy, only 8 patients were eligible.

## 3 RESULTS

**FIGURE 1 alz70375-fig-0001:**
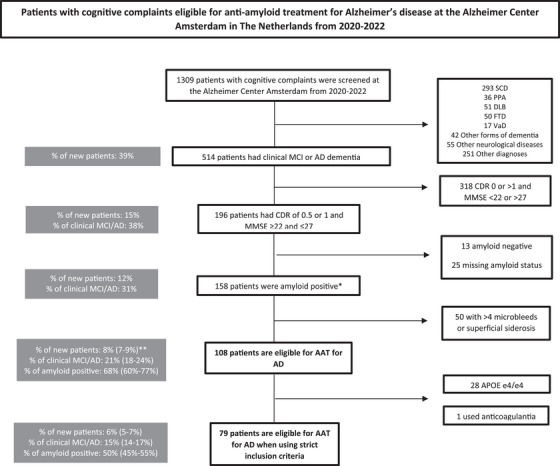
Clinical path of determining eligibility for anti‐amyloid treatment for Alzheimer's disease in patients with cognitive complaints at the Alzheimer Center Amsterdam in the Netherlands. *Amyloid status was based on amyloid PET (*n* = 39); when unavailable, CSF p‐tau/Aβ42 (*n* = 278) was used. Nineteen were amyloid positive and twenty were amyloid negative based on amyloid PET scan. Based on CSF p‐tau/Aβ42, 162 were amyloid positive and 116 were amyloid negative. ** Among patients with clinical MCI or AD, 25 had unknown amyloid status; we therefore calculated a range—assuming all 25 were amyloid positive (upper limit) or amyloid negative (lower limit)—with associated percentages shown in brackets. After excluding those with microbleeds or superficial siderosis, 13 patients remained eligible; their potential amyloid classifications (positive or negative) are likewise presented as a percentage range in brackets. AAT, anti‐amyloid treatment; Aβ, amyloid beta; AD, Alzheimer's disease; *APOE*, apolipoprotein E; CDR, Clinical Dementia Rating; CSF, cerebrospinal fluid; DLB, dementia with Lewy bodies; FTD, frontotemporal dementia; MCI, mild cognitive impairment; MMSE, Mini‐Mental State Examination; PET, positron emission tomography; PPA, primary progressive aphasia; p‐tau, phosphorylated tau; SCD, subjective cognitive decline; VaD, vascular dementia.

A total of 1309 patients (mean ± standard deviation age = 63 ± 8; MMSE = 25 ± 5) with cognitive concerns visited the memory clinic of Alzheimer Center Amsterdam for diagnostic workup between 2020 and 2022. Among these, 514 (39% of new patients) received a clinical diagnosis of MCI or AD (Figure 1). Of these 514 patients, 196 (15% of new patients and 38% of clinical MCI or AD) met inclusion criteria of having a CDR of 0.5 or 1.0 and a MMSE ≥ 22 and ≤ 27. Of these 196 patients, 158 (12% of new patients and 31% of clinical MCI or AD) were amyloid positive, while 13 were amyloid negative (1% of new patients and 3% of clinical MCI or AD) and 25 had a missing amyloid status (2% of new patients and 5% of clinical MCI or AD). Within the group of amyloid‐positive patients, 50 (32% of amyloid‐positive MCI/AD patients) had > 4 microbleeds or superficial siderosis, excluding them from further analysis. The remaining 108 (8% [range 7%–9% if missing amyloid status was taken into account] of new patients and 21% [18%–24%] of clinical MCI or AD) were eligible for AAT. After applying conservative criteria (excluding *APOE* ε4 homozygotes and those using anticoagulants), 79 (6% [5%–7%] of new patients and 15% [14%–17%] of clinical MCI or AD) remained eligible for AAT.

**RESEARCH IN CONTEXT**

1. **Systematic review**: Previous studies have explored the eligibility criteria for anti‐amyloid treatment (AAT) in Alzheimer's disease (AD), with estimates ranging from 1% to 18% of patients qualifying for treatment. However, real‐world data from clinical settings remain sparse.
2. **Interpretation**: Our findings suggest that a relatively small proportion of patients presenting to a tertiary memory clinic meet the eligibility criteria for AAT based on lecanemab's approved guidelines. This highlights potential barriers to treatment implementation, including stringent clinical and biomarker requirements. These results are consistent with previous projections but provide real‐world evidence from a standardized diagnostic cohort.
3. **Future directions**: Further research should examine how modifications to AAT eligibility criteria impact patient access, explore the use of alternative biomarkers to expand eligibility while ensuring safety, and identify strategies to enhance access to AAT in routine clinical practice.

## 4 DISCUSSION

This study shows that 8% (7%–9%) of new patients and 21% (18%–24%) of patients clinically diagnosed with MCI or AD who presented to our tertiary memory clinic between 2020 and 2022 would have been eligible for use of lecanemab. By including all consecutive patients from a tertiary memory clinic, we add real‐world evidence that is representative of the first settings in which lecanemab will be implemented, from Europe to prior data from simulation models and population‐based studies. Our estimates exceed those from a larger study using simulation models, which found that only 2% of new patients in six European countries (France, Germany, Italy, Spain, Sweden, and the United Kingdom) were eligible for AAT.^30^ A previous study in Sweden and Ireland reported somewhat comparable percentages of 7% and 9% of new patients to be eligible for AAT when following the appropriate use recommendations for aducanumab.^9^, ^11^ Recent data from the population‐based Mayo Clinic Study of Aging population (8% of all participants were eligible for lecanemab) is similarly in concordance with our results.^10^ Similarly, a study conducted in the Netherlands found a comparable eligibility rate of 8% for lecanemab.^16^ A study conducted at a geriatric outpatient clinic of a tertiary university hospital in Italy with a very high age and comorbidities found a marginal proportion of patients (0.7% of new patients) to be eligible for aducanumab, illustrating that high age and higher burden of comorbidities leads to a lower eligibility rate.^13^ This likely explains why we observed higher eligibility percentages in our cohort, which consists of a younger population with fewer comorbidities.[Fig alz70375-fig-0001]

Across all these studies, it is evident that a relatively low proportion of patients with a clinical diagnosis of MCI or AD are eligible for AAT, most likely due to strict eligibility criteria based on the risk of serious side effects. Use of a tertiary memory clinic population like ours has the advantage that this population is likely to be representative of the first settings in which AAT is implemented after it becomes available, but it has the potential downside of lacking generalizability to the general population. In this respect, it is reassuring that similar numbers were found in a population‐based setting.^10^ The limitations of the current study include a relatively young patient population with a low comorbidity burden. Another limitation is that we did not use all the in‐ and exclusion criteria according to the appropriate use recommendations (AUR) for lecanemab, for example, having an informant who can ensure that the patient has the support needed to be treated with lecanemab. Nevertheless, AURs are not intended to replace clinical judgment. Clinicians, in collaboration with patients and their care partners, make management decisions based on the patient's best interest, which may differ from the recommendations in AUR.

Together, the results of our study can be used as a source of information for the potential patient load expected when AAT is implemented in clinical practice. However, the actual capacity allocated will be determined by the final prescribing information for these drugs once approved; the willingness to receive AAT by patients; reimbursement levels; and agreements among regulators, payers, and providers. In the near future, costs of determining eligibility for AAT may be markedly reduced by use of blood‐based biomarkers for identification of amyloid positivity, as they are more scalable compared to CSF or amyloid PET.^31^ Especially in remote areas where CSF or amyloid PET scans are not part of the routine clinical use, a positive AD blood test might become a requirement to receive AAT. Eventually, these are all preparatory steps to ready society for a future with personalized medicine for AD with precise and molecular diagnosis and personalized risk profiles to target AD pathology more effectively.^32^

## 5 CONCLUSION

In our tertiary memory clinic, 8% (range 7%–9%) of new patients and 21% (range 18%–24%) of those clinically diagnosed with MCI or AD met in‐ and exclusion criteria for AAT. This information can be taken into account when estimating the preparedness of the health‐care system and budget‐impact analyses for these drugs.

## CONFLICT OF INTEREST STATEMENT

Sinthujah Vigneswaran, Frederik Barkhof, Afina W. Lemstra, and Yolande A.L. Pijnenburg have nothing to disclose. W.F., C.T., A.H., and E.V. are recipients of ABOARD, which is a public–private partnership receiving funding from ZonMw (73305095007) and Health∼Holland, Topsector Life Sciences & Health (PPP allowance, LSHM20106). Partners in ABOARD are Amsterdam UMC, locations VUmc and AMC, MUMC+, Erasmus MC, UMC Radboud, UMCG, TU Delft, Inholland, Vilans, Pharos, HealthRI, Jeroen Bosch Ziekenhuis, Medisch Centrum Leeuwarden, Zorg Innovatie Forum, Pharmo–STIZON, Alzheimer Nederland, Hersenstichting, KBO‐PCOB, PGGM, Zorgverzekeraars Nederland, CZ, Zilveren Kruis, Neurocast, Philips, ADx NeuroSciences, Castor, Vereniging Innovatieve Geneesmiddelen, Roche NL, Biogen NL, Novartis NL and the Brain Research Center. W.F., C.T., and E.G. are project leads on TAP‐Dementia, a ZonMw‐funded project (10510032120003) to optimize diagnosis of dementia, part of the Dutch National Dementia Strategy. W.F. is recipient of IHI‐PROMINENT project (grant agreement No. 101112145) and IHI‐AD‐RIDDLE project (grant agreement No. 101132933). PROMINENT and AD‐RIDDLE are supported by the Innovative Health Initiative Joint Undertaking (IHI JU). The JU receives support from the European Union's Horizon Europe research and innovation programme and COCIR, EFPIA, EuropaBio, MedTech Europe, and Vaccines Europe, with Davos Alzheimer's Collaborative, Combinostics OY, Cambridge Cognition Ltd., C2N Diagnostics LLC, and neotiv GmbH. Author disclosures are available in the supporting information.

## CONSENT STATEMENT

All human subjects provided written informed consent for use of biomaterials and medical data for the scientific research purposes presented in this article.

## Supporting information



Supporting Information
